# Association of Vitamin D and Weight Status With Neurodevelopmental Outcomes in a Large Pediatric Population: Cross-Sectional Study

**DOI:** 10.2196/89756

**Published:** 2026-02-27

**Authors:** Ying Shen, Meiying Gao, Lejing Guan, Lidan Sun, Yanfen Xu, Bethel Daniel, Kyla Zhang, Guannan Bai

**Affiliations:** 1 Children's Health Management Center Children’s Hospital Zhejiang University School of Medicine, National Clinical Research Center for Children and Adolescents' Health and Diseases Hangzhou China; 2 School of Public Health Brown University Providence, RI United States; 3 Warren Alpert Medical School Brown University Providence, RI United States; 4 Department of Child Health Care Children’s Hospital Zhejiang University School of Medicine, National Clinical Research Center for Children and Adolescents' Health and Diseases Hangzhou, Zhejiang China

**Keywords:** vitamin D, weight status, developmental outcomes, children, pediatrics

## Abstract

**Background:**

Vitamin D and weight status are key determinants for neurodevelopmental outcomes in children. However, their independent and interactive associations with neurodevelopmental outcomes represent a critical yet insufficiently explored issue in pediatric health.

**Objective:**

This study aims to evaluate the independent associations of vitamin D status and weight status with neurodevelopmental outcomes in a large pediatric cohort and to explore how these relationships are stratified by age and sex.

**Methods:**

This retrospective study analyzed the electronic health records of 19,283 healthy children who visited a health management center in a tertiary children’s hospital. Neurodevelopmental outcomes were assessed using the Ages and Stages Questionnaire, Third Edition (ASQ-3) (<6 years) and Conners Parent Rating Scale (CPRS) (≥6 years). Logistic regression analyses were applied to assess the independent and interactive impacts of vitamin D and weight status on neurodevelopmental outcomes.

**Results:**

The study included 19,283 children, 10,065 (52.2%) of whom were under 6 years old and 9218 (47.8%) who were between 6 and 18 years old. Among them, 1275 (12.67%) children under 6 were at risk for neurodevelopmental delay, and 1483 (16.09%) children aged between 6 and 18 years had behavioral challenges. Multivariable logistic regression revealed that vitamin D insufficiency/deficiency was significantly associated with suboptimal neurodevelopment in both the younger (odds ratio [OR] 1.48, 95% CI 1.23-1.79; *P*<.001) and older cohorts (OR 1.56, 95% CI 1.39-1.74; *P*<.001. A subgroup analysis revealed that vitamin D insufficiency/deficiency significantly increased the risk of neurodevelopmental delay among normal-weight girls under 6 years (OR 2.24, 95% CI 1.60-3.15; *P*<.001; *q*<.006) after the Benjamini-Hochberg false discovery rate correction. In the 6-to-18-year-old cohort, similar robust associations with behavioral challenges were observed in normal-weight girls (OR 1.61, 95% CI 1.30-2.00; *P*<.001; *q*<.006) and boys (OR 1.64, 95% CI 1.36-1.96; *P*<.001; *q*<.006).

**Conclusions:**

Vitamin D insufficiency/deficiency is associated with suboptimal neurodevelopmental outcomes in a sex-dependent manner modulated by weight status, challenging “one-size-fits-all” clinical models. We advocate for stratified, sex-specific screening and personalized pediatric care strategies.

## Introduction

Optimal neurodevelopment during childhood and adolescence is fundamental for lifelong cognitive function, emotional regulation, and social adaptation. However, this critical developmental window is increasingly threatened by two concurrent global public health challenges: the escalating prevalence of pediatric obesity and widespread vitamin D insufficiency [1,2]. The rising rates of childhood overweight and obesity, observed in both limited-income and high-income countries, including China, are no longer viewed solely as a risk for metabolic disease but also as a potential detriment to neurological health [3]. Concurrently, vitamin D, once primarily associated with skeletal homeostasis, is now recognized for its vital extraskeletal role, with a high prevalence of insufficiency and deficiency reported across diverse pediatric populations, independent of their nutritional status [4]. The potential intersection of these two prevalent conditions presents a significant and underinvestigated area of concern for pediatric health.

A substantial body of evidence has independently linked both adiposity and vitamin D status to neurodevelopmental outcomes [5-7]. Overweight and obesity in children are associated with chronic low-grade systemic inflammation and metabolic dysregulation, which can create a neuroinflammatory microenvironment detrimental to neural circuit maturation and synaptic plasticity [8]. On a parallel track, the biological plausibility for vitamin D's role in brain health is compelling. The vitamin D receptor and its activating enzymes are widely expressed throughout the developing brain, including in neurocritical regions like the hippocampus and prefrontal cortex [9]. As a potent neurosteroid, vitamin D modulates the expression of neurotrophic factors, regulates neurotransmitter synthesis, and exerts neuroprotective anti-inflammatory effects, making it indispensable for processes such as neuronal proliferation and synaptogenesis [9,10]. Clinical observations corroborate these mechanisms, with studies reporting lower serum vitamin D levels in children with neurodevelopmental disorders such as autism spectrum disorder (ASD) and attention-deficit/hyperactivity disorder (ADHD) compared to their neurotypical peers [11].

Despite the evidence establishing these separate associations, a critical knowledge gap exists in understanding their combined and interactive effects. Few large-scale epidemiological studies have simultaneously investigated vitamin D status, weight status, and neurodevelopment within a single pediatric cohort. This is a significant oversight, particularly given the intricate physiological relationship between these two factors; adiposity is a well-established risk factor for vitamin D deficiency, primarily through sequestration of this fat-soluble vitamin in adipose tissue, thereby reducing its systemic bioavailability [12]. Furthermore, much of the existing research is based on specific clinical populations (eg, children already diagnosed with neurodevelopmental disorders) or smaller samples, and there is a particular paucity of data from large, community-based cohorts of children spanning the full developmental spectrum from infancy to adolescence.

Given these research gaps, a comprehensive investigation in a large, diverse pediatric sample is warranted to investigate the complex interplay between vitamin D, weight status, and neurodevelopment. Therefore, this study aimed to investigate the associations of vitamin D status and weight status with neurodevelopmental outcomes in a large pediatric population aged between 0 and 18 years, using data from a major children’s health management center in East China.

## Methods

### Study Design and Participants

Our study retrospectively reviewed the electronic medical records database of participants who had health checkups at the Children's Hospital, Zhejiang University School of Medicine, from January 1, 2019, to June 30, 2025. This study included all children who met the following inclusion criteria: (1) aged ≤ 18 years old; (2) had data regarding serum levels of 25-hydroxyvitamin D, or 25(OH)D, and neurodevelopmental outcomes measured by the standard instruments; and (3) had no neurological disorders or physical disabilities. The exclusion criteria were as follows: (1) multiple records for one child, only retaining the earliest record; and (2) missing information on demographic characteristics such as age, sex, height, and weight.

### Ethical Considerations

This study was conducted in accordance with the Declaration of Helsinki [13] and was approved by the Medical Ethics Committee of the Children's Hospital, Zhejiang University School of Medicine (2021-IRB-185). Informed consent was waived by the ethics committee because the research involved a retrospective review of deidentified electronic health records and posed minimal risk to the participants. To ensure privacy and confidentiality, all data were fully deidentified and anonymized prior to analysis. No financial compensation or incentives were provided to the participants, as the data were collected during routine health checkups.

### Data Collection and Serum Levels 25(OH)D

Venous blood samples were collected from all participants in the morning after an overnight fast. Serum 25(OH)D levels were measured using a chemiluminescent immunoassay (25-Hydroxy Vitamin D Assay Kit, Orienter) on an automated analyzer. The laboratory strictly followed the manufacturer’s protocols, with regular internal quality control to ensure that the intraassay and interassay coefficients of variation were within clinical standards. According to the Global Consensus Recommendations on Prevention and Management of Nutritional Rickets [14], the sufficiency levels are defined as follows: sufficiency (>50 nmol/l), insufficiency (30-50 nmol/l), and deficiency (<30 nmol/l).

### Neurodevelopmental Outcomes

In alignment with standardized clinical screening protocols, neurodevelopmental outcomes were assessed using age-appropriate instruments. The Ages and Stages Questionnaire, Third Edition (ASQ-3) was employed for children under 6 years to capture early developmental milestones, while the Conners Parent Rating Scale (CPRS) was used for children aged between 6 and 18 years to evaluate behavioral and psychological symptoms. This transition between tools reflects the shifting clinical focus from motor and communication milestones in early childhood to behavioral and emotional regulation in school-aged children and adolescents. Age 6 serves as a critical clinical threshold in our health management center to ensure a streamlined workflow and avoid tool overlapping. The ASQ-3 was administered for neurodevelopmental screening in children under 6 years. This parent-completed tool, which takes approximately 10 to 15 minutes, consists of 30 items across 5 domains: communication, gross motor, fine motor, problem-solving, and personal-social. Each item is scored as 10 (yes), 5 (sometimes), or 0 (not yet). Domain-specific and total scores were calculated and compared to established age-specific cut-offs derived from the Chinese standardization of the ASQ-3 [15]. These Chinese norms were validated in a large-scale national sample to ensure cultural and linguistic appropriateness [15]. Children were categorized into 3 groups based on their scores relative to the mean (x) and standard deviation (SD): (1) “on schedule,” where scores were above x−1SD, indicating typical development; (2) “monitored,” where scores fell between x−2SD and x−1SD, indicating the monitoring zone; and (3) “possibly delayed,” where scores were ≤x−2SD, indicating a high risk of developmental delay. For this study, children in both the “monitored” and “possibly delayed” categories (ie, any score ≤x−1SD in one or more domains) were classified as being at risk for neurodevelopmental delay to ensure a comprehensive screening of children requiring clinical attention. The CPRS (Chinese version) was used to evaluate the behavioral and psychological status of children aged 3 to 17 years. Our study population’s actual age distribution fell entirely within its validated range; all participants were under the age of 18 (maximum age 17.9 years), and those aged ≥17.0 years represented a negligible fraction (54/19,283, 0.28%) of the total sample. Consequently, the use of the CPRS was both clinically appropriate and strictly aligned with its validated age parameters. The CPRS consists of 48 items rated on a 4-point Likert scale (0=not at all present to 3=very much present) and encompasses 5 subscales: oppositional (12 items), learning problems (4 items), psychosomatic problems (5 items), hyperactivity-impulsivity (4 items), and anxiety (4 items) [16]. The score for each subscale was determined by the mean of its component items. A participant was classified as having behavior problems if one or more subscale scores exceeded the Chinese standardized norms (ie, the mean score by more than two SDs) [17]. The questionnaires were completed by parents or primary guardians under the supervision of trained health professionals.

### Covariate Evaluation

The selection of covariates for the multivariable models was primarily determined by the availability of variables in our health management database, further guided by clinical expertise and established literature on pediatric neurodevelopment. Consequently, we included all available clinical and demographic parameters from the electronic health records that were identified as potential confounders, including age, sex, and weight status. Age was calculated as the time interval from birth to the examination date. Height and weight were measured using the age-appropriate equipment and protocols. For children under 3 years of age, recumbent length was measured in the supine position using a baby height-measuring board, while for children aged 3 years and older, standing height was measured using a calibrated stadiometer. Weight was measured to the nearest 50 g using an infant electronic scale for children under 2 years of age and a lever scale for those older than 2 years. All measurements were taken with the children wearing minimal clothing and no shoes. Weight status across all age groups was classified according to the World Health Organization (WHO) growth standards [18]. Weight status for children under 2 years was defined according to weight-for-length *z* score criteria. Participants were classified as underweight (*z* score < –2), overweight (*z* score > 2), and obesity (*z* score > 3). For children and adolescents aged 2 to 18 years, BMI was calculated as weight (kg) divided by height squared (m^2^). In accordance with WHO standards, participants were classified as underweight (BMI for age ≤5th percentile), overweight (≥85th percentile), or obese (≥95th percentile) [18].

### Statistical Analysis

A complete case analysis was performed for this study. Participants with missing values for serum 25(OH)D, neurodevelopmental screening scores, or covariates (age, sex, and weight status) were excluded during the initial data cleaning phase, as detailed in our inclusion and exclusion criteria. This resulted in a final analytic sample of 19,283 children with complete information for all variables of interest. First, the normality of continuous variables was assessed using the Kolmogorov-Smirnov test. In this study, all continuous variables followed a normal distribution; therefore, the results are presented as mean and SD. Categorical variables are expressed as numbers and percentages. Differences in the distribution of the above dependent variables were compared between children with and without typical neurodevelopmental outcomes using *t* tests for continuous variables and chi-square tests for categorical variables. In addition, we assessed sex differences in weight status and vitamin D nutritional status in the 2 age groups (between 0 and 6 years and above 6 years). Because some vitamin D deficiency subgroups had small sample sizes, we combined the insufficiency and deficiency categories for the following analysis. Second, we evaluated the associated factors for suboptimal neurodevelopmental outcomes in the study population using binary logistic regression analysis. Further, a subgroup analysis was conducted using the binary logistic regression models to assess the association between vitamin D nutritional status and neurodevelopmental outcomes across different subgroups of weight status in both boys and girls. To account for the increased risk of type I errors due to multiple comparisons, we applied the Benjamini-Hochberg false discovery rate (FDR) correction. The findings were considered statistically robust if the FDR-adjusted *q* value was <.05. For results where the original *P* value was <.05 but the *q* value fell between .05 and .10, the associations were interpreted as “marginally significant” or “suggestive trends” rather than definitive findings, ensuring a more conservative and prudent interpretation of our results.

All statistical analyses were conducted using SPSS Statistics (version 27.0; IBM Corp) and R software (version 4.4.1; R Foundation for Statistical Computing). Statistical significance was indicated when *P*<.05 (2 tails).

## Results

### General and Clinical Characteristics of the Study Population

Table 1 outlines the general and clinical characteristics of the study population. The study analyzed a total of 10,065 (52.2%) children aged below 6 years and 9218 (47.8%) children aged 6 years or above. Among children under the age of 6 years, 1275 (12.67%) were at risk for neurodevelopmental delay. Among children older than 6 years, 1483 (16.09%) were identified to have behavioral challenges. In both age groups, children with suboptimal neurodevelopmental outcomes (ie, being at risk for neurodevelopmental delay under age 6 and having behavioral challenges older than 6) were significantly younger, more often boys, and had higher BMI and lower 25(OH)D level (all *P* values <.05).

**Table 1:** General and clinical characteristics of the study population (N=19,283).

Characteristics		<6 years					≥6 years				
		Overall (n=10,065)	Typicality (n=8790)	At risk for delay (n=1275)	*P* value		Overall (n=9218)	Typicality (n=7,735)	Having behavioral challenges (n=1,483)	*P* value	
Age (years), mean (SD)		2.76 (1.57)	2.81 (1.56)	2.48 (1.59)	<.001		9.76 (2.61)	9.79 (2.63)	9.58 (2.52)	.003	
**Sex, n (%)**											<.001
	Male	5794 (57.57)	4909 (55.85)	885 (69.41)	<.001		5771 (62.61)	4903 (63.39)	868 (58.53)		
	Female	4271 (42.43)	3881 (44.15)	390 (30.59)			3447 (37.39)	2832 (36.61)	615 (41.47)		
Height (cm), mean (SD)		92.32 (14.73)	92.78 (14.60)	89.15 (15.24)	<.001		140.30 (16.37)	140.66 (16.49)	138.42 (15.60)	<.001	
Weight (kg), mean (SD)		13.58 (3.98)	13.69 (3.98)	12.86 (3.90)	<.001		35.24 (14.61)	35.52 (14.72)	33.80 (13.92)	<.001	
BMI (kg/m^2^), mean (SD)		15.77 (1.71)	15.74 (1.68)	16.03 (1.86)	<.001		17.18 (3.66)	17.22 (3.66)	16.96 (3.61)	.01	
**Weight status, n (%)**											.05
	Underweight	834 (8.29)	723 (8.23)	111 (8.71)	.46		781 (8.47)	633 (8.18)	148 (9.98)		
	Normal weight	8521 (84.66)	7456 (84.82)	1065 (83.53)			6299 (68.33)	5290 (68.39)	1009 (68.04)		
	Overweight and obesity	710 (7.05)	611 (6.95)	99 (7.76)			2138 (23.19)	1812 (23.43)	326 (21.98)		
25(OH)D^a^ (nmol/L), mean (SD)		98.37 (42.76)	98.82 (42.58)	95.28 (43.89)	.006		56.69 (23.47)	57.47 (23.78)	52.63 (21.33)	<.001	
**Vitamin D nutritional status, n (%)**											<.001
	Sufficiency	8954 (88.96)	7841 (89.20)	1113 (87.29)	.08		5020 (54.46)	4333 (56.02)	687 (46.33)		
	Insufficiency	1021 (10.14)	875 (9.95)	146 (11.45)			3422 (37.12)	2766 (35.76)	656 (44.23)		
	Deficiency	90 (0.89)	74 (0.84)	16 (1.25)			776 (8.42)	636 (8.22)	140 (9.44)		

^a^25(OH)D: 25-hydroxyvitamin D.

In addition, Multimedia Appendices 1 and 2 highlight the sex differences in the proportion of weight status and vitamin D nutritional status in the two age groups. Girls at risk for neurodevelopmental delay had significantly higher rates of vitamin D insufficiency/deficiency than their peers with typical neurodevelopment (15.64% vs 11.00%; *P*=.006). In children above 6 years old, we observe significantly higher rate of vitamin D insufficiency/deficiency in both boys and girls with behavioral challenges compared to their typical peers (boys: 50.35% vs 42.12%; *P*<.001 and girls: 58.37% vs 47.21%; *P*<.001). Multimedia Appendices 3 and 4 present the rates of being at risk for neurodevelopmental delay (<6 years) and behavioral challenges (>6 years) across the subgroups of weight status and vitamin D nutritional status, respectively. The rate of being at risk for neurodevelopmental delay was highest in the subgroup of underweight + vitamin D insufficiency/deficiency (18.75%), followed by that in the subgroup of overweight/obesity + vitamin D insufficiency/deficiency (17.95%). The rate of behavioral challenges was highest in the subgroup of underweight + vitamin D insufficiency/deficiency (23.7%), followed by that in the subgroup of normal weight + vitamin D insufficiency/deficiency (19.38%), while the rate in the subgroup of overweight/obesity + vitamin D insufficiency/deficiency was relatively low (16.61%).

### Associated Factors of Suboptimal Neurodevelopmental Outcomes by Multivariable Logistic Regression Analysis

Table 2 presents the results of the logistic regression analysis regarding the associated factors of being at risk of neurodevelopmental delay in children under 6 years old. Older age, being male, and having vitamin D insufficiency/deficiency were statistically significantly associated with being at risk for neurodevelopmental delay (all *P* values <.05). More specifically, when a child had vitamin D insufficiency or deficiency, the odds of being at risk of neurodevelopmental delay were 1.48 times higher than those who had sufficient vitamin D (95% CI 1.23-1.79; *P*<.001).

**Table 2:** Logistic regression analysis of factors associated with risk of neurodevelopmental delay in children under 6 years of age (n=10,065).

Characteristics		OR (95% CI)	*P* value
Age		0.84 (0.81-0.88)	<.001
**Sex**			
	Female	reference	N/A^a^
	Male	1.79 (1.57-2.03)	<.001
**Weight status**			
	Normal weight	reference	N/A
	Underweight	1.23 (0.99-1.53)	.06
	Overweight and obesity	1.20 (0.96-1.50)	.12
**Vitamin D nutritional status**			
	Sufficiency	reference	N/A
	Insufficiency/Deficiency	1.48 (1.23-1.79)	<.001

^a^N/A: not applicable.

The associated factors for each specific domain of ASQ had different patterns (Multimedia Appendix 5). To be noted, underweight and overweight/obesity were significantly associated with delays in gross motor (underweight: OR 1.58, 95% CI 1.17-2.12; *P*=.003; overweight/obesity: OR 1.64, 95% CI 1.21-2.23; *P*=.002) and fine motor (underweight: OR 1.77, 95% CI 1.32-2.38; *P*<.001; overweight/obesity: OR 1.55, 95% CI 1.13-2.14; *P*=.007). Vitamin D insufficiency/deficiency was significantly associated with elevated odds of being at risk of delay at the overall level and each domain level (all *P*<.05). There was also a sex difference regarding the pattern of associated factors, as underscored in Multimedia Appendices 6 and 7. Boys with overweight/obesity had a higher risk of overall delay (odds ratio [OR] 1.30, 95% CI 1.01-1.68; *P*=.042), while vitamin D insufficiency or deficiency was not statistically significantly associated with overall delay or delay in a specific domain except for communication (OR 1.57, 95% CI 1.02-2.42; *P*=.042). However, in girls, the significant association between overweight/obesity and delay disappeared, and vitamin D insufficiency or deficiency had a statistically significant association with overall delay or delay in a specific domain (*P*<.05).

Table 3 presents the results of the logistic regression analysis in terms of the associated factors of having behavioral challenges in children above 6 years old. Older age, being female, being underweight, and having vitamin D insufficiency/deficiency were statistically significantly associated with having behavioral challenges (all *P*<.05). More specifically, vitamin D insufficiency or deficiency was significantly associated with an increased odds of having behavioral challenges (OR 1.56, 95% CI 1.39-1.74; *P*<.001).

**Table 3:** Logistic regression analysis of factors associated with behavioral challenges in children over 6 years (n=9218).

Characteristics		OR (95% CI)	*P* value
Age		0.95 (0.93-0.98)	<.001
**Sex**			
	Female	reference	N/A^a^
	Male	1.19 (1.06-1.33)	.003
**Weight status**			
	Normal weight	reference	N/A
	Underweight	1.25 (1.04-1.52)	.02
	Overweight and obesity	0.95 (0.83-1.10)	.50
**Vitamin D nutritional status**			
	Sufficiency	reference	N/A
	Insufficiency/deficiency	1.56 (1.39-1.74)	<.001

^a^N/A: not applicable.

The pattern of associated factors for each domain of CPRS differed (Multimedia Appendix 8). In particular, underweight was significantly associated with elevated risk of having psychosomatic problems (OR 1.31, 95% CI 1.01-1.71; *P*=.043), while overweight/obesity was significantly associated with lower odds of having psychosomatic problems (OR 0.68, 95% CI 0.55-0.85; *P*<.001). Vitamin D insufficiency/deficiency was significantly associated with elevated odds of behavioral challenges and odds of oppositional disorder, learning challenges, psychosomatic challenges, and hyperactivity-impulsivity at the domain level (all *P*<.05). Multimedia Appendices 9 and 10 display the profiles of associated factors in boys and girls, respectively.

### Subgroup Analysis of the Association Between Vitamin D and Neurodevelopmental Outcomes Across Different Weight Subgroups

**Figure 1:**
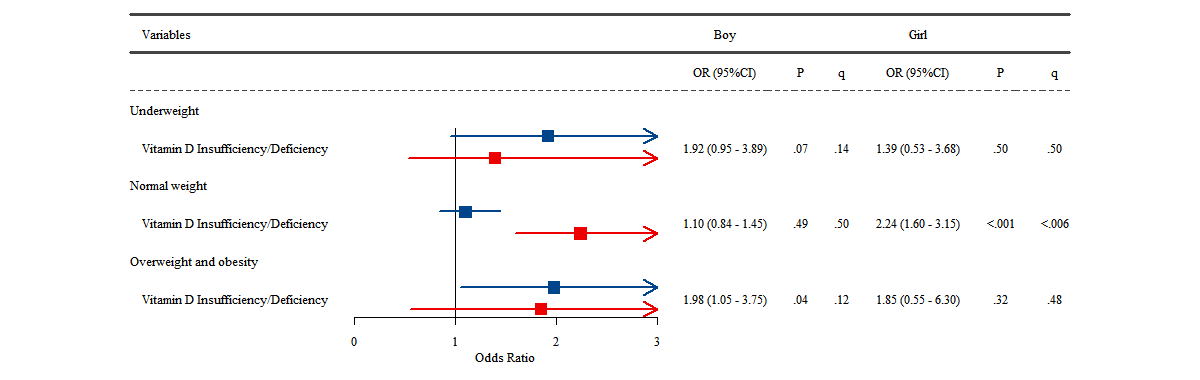
Associations between Vitamin D status and being at risk for neurodevelopmental delay in children aged under 6 years, stratified by sex and weight status.

**Figure 2:**
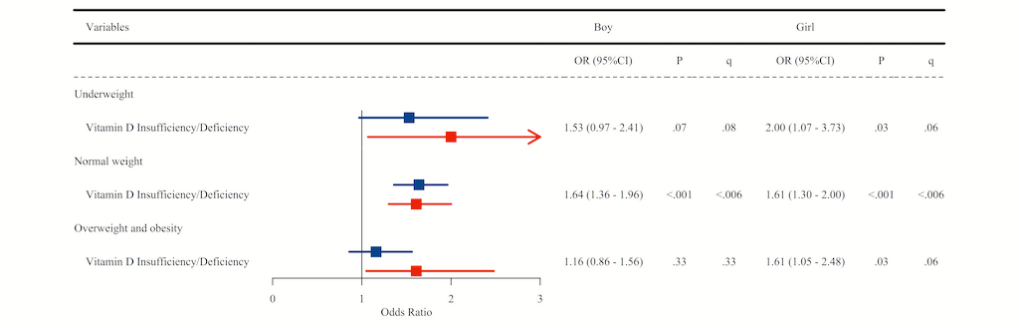
Associations between Vitamin D status and behavior problems in children aged above 6 years, stratified by sex and weight status.

Figure 1 contains a forest plot showing the associations between vitamin D insufficiency/deficiency and neurodevelopmental delay in boys and girls under 6 years old in the underweight, normal weight, and overweight/obesity subgroups. Notably, after FDR correction, the most robust association was observed in the normal-weight subgroup, where girls with vitamin D insufficiency/deficiency had a significantly higher risk of neurodevelopmental delay (OR 2.24, 95% CI 1.60-3.15; *P*<.001, q<.006). Figure 2 demonstrates the associations between vitamin D insufficiency/deficiency and behavioral challenges in boys and girls above 6 years old in the underweight, normal weight, and overweight/obesity subgroups. Among girls, while the original *P* values indicated significant associations before FDR corrections across all weight categories (all *P*<.05), these associations remained statistically robust primarily in the normal-weight group (OR 1.61, 95% CI 1.30-2.00; *P*<.001; *q*<.006) and showed a marginal significance in the underweight and overweight/obesity groups (all *q*=.06). Among boys, the association remained significant only in the normal-weight subgroup, where vitamin D insufficiency/deficiency was linked to a higher chance of behavioral challenges (OR 1.64, 95% CI 1.36-1.96; *P*<.001; *q*<.006).

## Discussion

### Principal Findings

This large-scale study revealed the complex relationship of vitamin D and weight status with neurodevelopmental outcomes across a wide pediatric age range. Our principal and most significant finding is a profound sex-specific divergence in risk profiles: in children under 6 years, neurodevelopmental risk in boys was predominantly linked to overweight/obesity, whereas in girls, vitamin D insufficiency/deficiency emerged as the critical independent determinant. Furthermore, we uncovered an unexpected pattern in that the harmful effects of low vitamin D on neurodevelopment were surprisingly strongest in normal-weight children. Collectively, these findings challenge a simplistic risk model, suggesting that the pathways linking vitamin D and weight status to childhood neurodevelopment are fundamentally moderated by both sex and developmental context. This highlights the necessity for more tailored, sex- and context-specific strategies in both future research and clinical practice.

Our study first highlights the significant public health challenge of vitamin D insufficiency in Chinese children, revealing a dramatic age-related disparity. While the combined prevalence of vitamin D insufficiency and deficiency was 11.03% in children under 6 years, this figure notably increased to 45.54% in the 6-to-18 years age group. This significant increase is consistent with other reports from China [19] and likely reflects a critical gap in public health practices. The current supplementation guidelines and parental attention are predominantly focused on infancy and early childhood [20,21], after which routine supplementation often stops [22]. The prevalence in our cohort, particularly among the older group, appears considerably higher than that reported in children in the United States by the National Health and Nutrition Examination Survey (2011-2014) [23], underscoring the urgent need to revise and extend public health strategies in China.

A main finding of our study is the confirmation that vitamin D insufficiency/deficiency is a strong independent risk factor for suboptimal neurodevelopmental outcomes, after adjusting for a comprehensive set of critical covariates, including age, sex, and weight status. What makes this association particularly compelling is the distinct and pervasive nature of the risk profile associated with poor vitamin D status when compared to that of weight status.

Our analysis revealed that the impact of abnormal weight status was primarily domain-specific and manifested differently across developmental stages. While it was associated with gross and fine motor skill delays in the younger cohort (under 6 years), it was linked to psychosomatic disorders in the older cohort (between 6 and 18 years). These divergent patterns likely reflect both age-dependent biological vulnerabilities and the distinct clinical focus of the assessment tools optimized for each age group.

This association likely reflects different physiological mechanisms depending on weight status: underweight children may lack essential nutrients needed for brain growth, while those with overweight or obesity may experience chronic inflammation and metabolic disruption. In contrast, vitamin D insufficiency/deficiency demonstrated a remarkably global effect that transcended developmental stages. In our younger cohort, this manifested as pan-domain developmental delays, while in the older group, it presented as broad-spectrum behavioral and learning challenges. This broad-spectrum risk profile not only aligns with but also substantially extends the scope of previous research, which often linked low vitamin D to more isolated outcomes such as communication delays or an increased risk for ADHD [24,25]. The consistency of this widespread risk reinforces the hypothesis that vitamin D functions as a fundamental neurosteroid, a role supported by strong biological plausibility. Vitamin D acts via receptors that are ubiquitously expressed throughout the brain, where it modulates genes controlling fundamental neurodevelopmental processes, including neuronal differentiation, synaptic plasticity, and neuroprotection [9,11,26-30]. A deficit in this essential nutrient can thus lead to widespread disruption at any stage from early brain formation to later neural maturation. This highlights vitamin D deficiency as a fundamental threat to brain health throughout childhood.

Beyond the aforementioned main effects, our study reveals a complex, sex-specific interplay between vitamin D status and weight status that challenges simplistic, additive risk models. Following rigorous FDR correction, the most robust associations between vitamin D insufficiency/deficiency and adverse neurodevelopmental outcomes were consistently observed within the normal-weight subgroup across all age groups in girls and among boys aged 6 years and older. In normal-weight children, the absence of major metabolic disturbances—often associated with extreme weight statuses—may provide a clearer “biological window” to observe the direct neurotropic impact of vitamin D. This suggests that when weight status is within the normal range, the brain’s foundational development and behavioral regulation may be more directly vulnerable to the lack of this critical neurosteroid.

In children with overweight or obesity, neurodevelopment is influenced by a multifactorial array of risks, including chronic inflammation and metabolic dysregulation, which may independently impair cognitive and behavioral functions. These competing risks may “mask” or dilute the specific contribution of vitamin D in our statistical models, leading to the attenuated associations observed (*q*>.05). Interestingly, in girls aged 6 years and older, a consistent but marginally significant trend was observed in the underweight and overweight/obesity subgroups (both *q*=.06). This suggests that the neuroprotective role of vitamin D in girls above 6 years may be more pervasive across different body compositions than in other cohorts. The attenuation from significance (*P*<.05) to a marginal trend (*q*=.06) after FDR correction likely reflects the reduced statistical power within these smaller samples of weight-extreme strata, rather than a true absence of biological effects. Nevertheless, these findings should be considered exploratory, warranting further validation in even larger specialized cohorts of pediatric populations with underweight or overweight/obesity.

Our findings urge a shift toward a more precise, stratified approach in neurodevelopmental surveillance. From a public health perspective, the sharp rise in vitamin D inadequacy after infancy highlights a critical gap in health education, necessitating revised strategies that advocate for continued supplementation throughout childhood. In the clinical setting, our results call for more proactive and risk-based screening. Our data suggest that clinicians should view the combination of weight status and vitamin D insufficiency/deficiency as an identifiable risk signal for future neurodevelopmental and behavioral adversity. Our results suggest that girls may exhibit greater vulnerability to the neurodevelopmental impacts of vitamin D insufficiency/deficiency. For girls older than 6 years, the risks associated with vitamin D insufficiency/deficiency should be taken seriously, regardless of their weight status.

### Strengths and Limitations

Our study has several key strengths. The main strength is its large sample size, which ensures sufficient statistical power and allows us to examine complex sex-specific relationships that differ between boys and girls. This kind of detailed analysis is often not possible in smaller studies. Second, the inclusion of a wide age range, combined with validated, age-appropriate neurodevelopmental assessment tools (ASQ-3 and CPRS), was essential for revealing how these risk factors manifest differently across distinct developmental stages. Furthermore, our rigorous adjustment for a comprehensive set of covariates strengthens the conclusion that vitamin D status and weight status are independently associated with neurodevelopmental outcomes.

Nevertheless, we must acknowledge several limitations. First, the cross-sectional design of this study establishes associations but precludes definitive causal inference. We cannot entirely rule out reverse causality, where children with subtle neurodevelopmental or behavioral challenges may have reduced outdoor activities or restrictive dietary habits, leading to secondary vitamin D deficiency. However, since our participants were primarily from the general pediatric population undergoing routine health screenings, the impact of severe clinical behaviors is likely limited. Future longitudinal studies are necessary to elucidate this potentially bidirectional relationship. Second, our outcomes were based on parent-reported screening questionnaires. While these tools are highly feasible for large-scale epidemiological research, they are not a substitute for gold-standard clinical diagnoses and may be subject to reporting bias. Third, despite the adjustment for essential clinical covariates, the potential for residual confounding remains. As our data were derived from routine electronic health records rather than structured research surveys, certain socioenvironmental and lifestyle factors, such as parental education level, family income, and outdoor physical activity, were not available in our database. These factors are known to influence both vitamin D levels and neurodevelopmental outcomes. Although we included all relevant clinical variables available in our records to mitigate this, our results should be interpreted with caution regarding these unmeasured confounders. Fourth, our study utilized different assessment tools for the two age cohorts (ASQ-3 and CPRS). While both are validated for their respective populations, their domains do not completely overlap (eg, hyperactivity is not captured by ASQ-3). Consequently, our findings should be interpreted as stage-specific associations rather than a continuous developmental trajectory across the pediatric lifespan.

### Conclusions

Our study provides compelling evidence that vitamin D deficiency is an independent determinant of neurodevelopmental and behavioral adversities throughout childhood. The interaction with weight status is not uniform but sex dependent. These findings highlight the inadequacy of a one-size-fits-all approach. We advocate for a shift toward proactive, stratified screening, where neurodevelopmental risk is assessed through a dynamic, sex- and context-specific lens to enable truly personalized pediatric care.

## Appendix

Multimedia Appendix 1 Sex differences in the proportion of weight status and vitamin D nutritional status in children between 0 and 6 years old with typical neurodevelopment and those at risk for delay (n=10,065).

Multimedia Appendix 2 Sex differences in the proportion of weight status and vitamin D nutritional status in children above 6 years old with and without behavioral challenges (n=9218).

Multimedia Appendix 3 Rates of being at risk for neurodevelopmental delay in subgroups of weight status and vitamin D nutritional status in children under 6 years old (n=10,065).

Multimedia Appendix 4 Rates of behavior problems in subgroups of weight status and vitamin D nutritional status in children above 6 years old (n=9218).

Multimedia Appendix 5 Associated factors of different ASQ-3 (Ages and Stages Questionnaire, Third Edition) domains in children under 6 years old using logistic regression analysis (n=10,065).

Multimedia Appendix 6 Associated factors of being at risk of neurodevelopmental delay in boys under 6 years old using logistic regression analysis (n=5794).

Multimedia Appendix 7 Associated factors of being at risk of neurodevelopmental delay in girls under 6 years old according the logistic regression analysis (n=4271).

Multimedia Appendix 8 Associated factors of different Conners Parent Rating Scale (CPRS) subscales in children above 6 years via logistic regression analysis (n=9218).

Multimedia Appendix 9 Associated factors of behavioral challenges in boys above 6 years using logistic regression analysis (n=5771).

Multimedia Appendix 10 Associated factors of behavioral challenges in girls above 6 years using logistic regression analysis (n=3447).

Multimedia Appendix 11 STROBE (Strengthening the Reporting of Observational Studies in Epidemiology): checklist of items that should be included in reports of observational studies.

## Abbreviations

25(OH)D: 25-hydroxyvitamin D
ADHD: attention-deficit/hyperactivity disorder
ASD: autism spectrum disorder
ASQ-3: Ages and Stages Questionnaire, Third Edition
CPRS: Conners Parent Rating Scale
OR: odds ratio
WHO: World Health Organization

## Data Availability

The data that support the findings of this study are available from the corresponding author (guannanbai@zju.edu.cn) upon reasonable request.
