# Supplementary material for: Association of Vitamin D and Weight Status With Neurodevelopmental Outcomes in a Large Pediatric Population: Cross-Sectional Study
Source: JMIR Public Health Surveill. 2026 Feb 27;12:e89756. doi: 10.2196/89756 (PMC12988349; doi:10.2196/89756)
Supplement: Multimedia Appendix 1 [file publichealth_v12i1e89756_app1.docx]

Multimedia Appendix 1: Sex differences in the proportion of weight status and Vitamin D nutritional status in 0-6 year old children between children with typical neurodevelopment and those being at risk for delay (n=10,065).

| Characteristics | Boys (n=5,794) | | | Girls (n=4,271) | | |
| --- | --- | --- | --- | --- | --- | --- |
|  | Typicality (n=4,909) | At risk for delay (n=885) | *P* value | Typicality (n=3,881) | At risk for delay (n=390) | *P* value |
| Weight status, n (%) |  |  |  |  |  |  |
| Underweight | 442 (9.00) | 81 (9.15) | .37 | 281 (7.24) | 30 (7.69) | .50 |
| Normal weight | 4080 (83.11) | 722 (81.58) |  | 3376 (86.99) | 343 (87.95) |  |
| Overweight and obesity | 387 (7.88) | 82 (9.27) |  | 224 (5.77) | 17 (4.36) |  |
| Vitamin D nutritional status, n (%) |  |  |  |  |  |  |
| Sufficiency | 4387 (89.37) | 784 (88.59) | .49 | 3454 (89.00) | 329 (84.36) | .006 |
| Insufficiency/Deficiency | 522 (10.63) | 101 (11.41) |  | 427 (11.00) | 61 (15.64) |  |

Note: Data are presented as n (%). P-values were calculated using the Chi-square test to compare the distribution of weight status and vitamin D nutritional status between children with typical neurodevelopment and those at risk for delay, stratified by sex. Statistical significance was defined as P < .05.
