# Supplementary material for: Association of Vitamin D and Weight Status With Neurodevelopmental Outcomes in a Large Pediatric Population: Cross-Sectional Study
Source: JMIR Public Health Surveill. 2026 Feb 27;12:e89756. doi: 10.2196/89756 (PMC12988349; doi:10.2196/89756)
Supplement: Multimedia Appendix 2 [file publichealth_v12i1e89756_app2.docx]

**Multimedia Appendix 2:** Sex differences in the proportion of weight status and Vitamin D nutritional status children above 6 years old with and without behavior problems (n=9,218).

| Characteristics | Boys(n=5,771) | | | Girls (n=3,447) | | |
| --- | --- | --- | --- | --- | --- | --- |
|  | Typicality (n=4,903) | Having behavior problems (n=868) | *P* value | Typicality (n=2,832) | Having behavior problems (n=615) | *P* value |
| Weight status, n (%) |  |  |  |  |  |  |
| Underweight | 412 (8.40) | 96 (11.06) | .02 | 221 (7.80) | 52 (8.46) | .74 |
| Normal weight | 3190 (65.06) | 562 (64.75) |  | 2100 (74.15) | 447 (72.68) |  |
| Overweight and obesity | 1301 (26.53) | 210 (24.19) |  | 511 (18.04) | 116 (18.86) |  |
| Vitamin D nutritional status, n (%) |  |  |  |  |  |  |
| Sufficiency | 2838 (57.88) | 431 (49.65) | <.001 | 1495 (52.79) | 256 (41.63) | <.001 |
| Insufficiency/Deficiency | 2065 (42.12) | 437 (50.35) |  | 1337 (47.21) | 359 (58.37) |  |

Note: Data are presented as n (%). P values were calculated using the Chi-square test to compare the distribution of weight status and vitamin D nutritional status between children with and without behavior problems, stratified by sex. All reported P values are two-sided, and statistical significance was defined as P<.05.
