# Supplementary material for: Association of Vitamin D and Weight Status With Neurodevelopmental Outcomes in a Large Pediatric Population: Cross-Sectional Study
Source: JMIR Public Health Surveill. 2026 Feb 27;12:e89756. doi: 10.2196/89756 (PMC12988349; doi:10.2196/89756)
Supplement: Multimedia Appendix 3 [file publichealth_v12i1e89756_app3.docx]

**Multimedia Appendix 3:** Rates of being at risk for neurodevelopmental delay in subgroups of weight status and vitamin D nutritional status in children under 6 years old (n=10,065).

| Weight status | Vitamin D nutritional status | Overall (n=10,065) | | Boys (n=5,794) | | Girls (n=4,271) | |
| --- | --- | --- | --- | --- | --- | --- | --- |
|  |  | Typicality | At risk for delay | Typicality | At risk for delay | Typicality | At risk for delay |
| Underweight | Sufficiency | 645 (87.40) | 93 (12.60) | 403 (85.38) | 69 (14.62) | 242 (90.98) | 24 (9.02) |
|  | Insufficiency/Deficiency | 78 (81.25) | 18 (18.75) | 39 (76.47) | 12 (23.53) | 39 (86.67) | 6 (13.33) |
| Normal weight | Sufficiency | 6681 (87.64) | 942 (12.36) | 3653 (84.89) | 650 (15.11) | 3028 (91.20) | 292 (8.80) |
|  | Insufficiency/Deficiency | 775 (86.30) | 123 (13.70) | 427 (85.57) | 72 (14.43) | 348 (87.22) | 51 (12.78) |
| Overweight and obesity | Sufficiency | 515 (86.85) | 78 (13.15) | 331 (83.59) | 65 (16.41) | 184 (93.40) | 13 (6.60) |
|  | Insufficiency/Deficiency | 96 (82.05) | 21 (17.95) | 56 (76.71) | 17 (23.29) | 40 (90.91) | 4 (9.09) |

Note: Data are presented as numbers (percentages). The numbers in parentheses within the table represent the row percentages, as specified.
