# Supplementary material for: Association of Vitamin D and Weight Status With Neurodevelopmental Outcomes in a Large Pediatric Population: Cross-Sectional Study
Source: JMIR Public Health Surveill. 2026 Feb 27;12:e89756. doi: 10.2196/89756 (PMC12988349; doi:10.2196/89756)
Supplement: Multimedia Appendix 4 [file publichealth_v12i1e89756_app4.docx]

**Multimedia Appendix 4:** Rates of behavior problems in subgroups of weight status and vitamin D nutritional status in children above 6 years old (n=9,218).

| Weight status | Vitamin D nutritional status | Overall (n=9,218) | | Boys (n=5,771) | | Girls(n=3,447) | |
| --- | --- | --- | --- | --- | --- | --- | --- |
|  |  | Typicality | Having behavior problems | Typicality | Having behavior problems | Typicality | Having behavior problems |
| Underweight | Sufficiency | 398 (84.14) | 75 (15.86) | 270 (83.33) | 54 (16.67) | 128 (85.91) | 21 (14.09) |
|  | Insufficiency/Deficiency | 235 (76.30) | 73 (23.70) | 142 (77.17) | 42 (22.83) | 93 (75.00) | 31 (25.00) |
| Normal weight | Sufficiency | 3052 (86.63) | 471 (13.37) | 1930 (87.37) | 279 (12.63) | 1122 (85.39) | 192 (14.61) |
|  | Insufficiency/Deficiency | 2238 (80.62) | 538 (19.38) | 1260 (81.66) | 283 (18.34) | 978 (79.32) | 255 (20.68) |
| Overweight and obesity | Sufficiency | 883 (86.23) | 141 (13.77) | 638 (86.68) | 98 (13.32) | 245 (85.07) | 43 (14.93) |
|  | Insufficiency/Deficiency | 929 (83.39) | 185 (16.61) | 663 (85.55) | 112 (14.45) | 266 (78.47) | 73 (21.53) |

Note: Data are presented as numbers (percentages). The numbers in parentheses within the table represent the row percentages, as specified.
