# Supplementary material for: Association of Vitamin D and Weight Status With Neurodevelopmental Outcomes in a Large Pediatric Population: Cross-Sectional Study
Source: JMIR Public Health Surveill. 2026 Feb 27;12:e89756. doi: 10.2196/89756 (PMC12988349; doi:10.2196/89756)
Supplement: Multimedia Appendix 5 [file publichealth_v12i1e89756_app5.docx]

**Multimedia Appendix 5:** Associated factors of different ASQ-3 domains in children under 6 years old by the logistic regression analysis (n=10,065).

| Characteristics | Communication | | Gross Motor | | Fine Motor | | Problem Solving | | Personal-Social | |
| --- | --- | --- | --- | --- | --- | --- | --- | --- | --- | --- |
|  | OR (95%CI) | *P* value | OR (95%CI) | *P* value | OR (95%CI) | *P* value | OR (95%CI) | *P* value | OR (95%CI) | *P* value |
| Age | 0.65 (0.59-0.71) | <.001 | 0.99 (0.93-1.05) | .66 | 0.87 (0.82-0.93) | <.001 | 0.67 (0.61-0.74) | <.001 | 0.87 (0.81-0.93) | <.001 |
| Sex |  |  |  |  |  |  |  |  |  |  |
| Girl | reference |  | reference |  | reference. |  | reference |  | reference |  |
| Boy | 2.35 (1.81-3.03) | <.001 | 1.32 (1.09-1.61) | .004 | 2.15 (1.75-2.65) | <.001 | 1.59 (1.20-2.10) | .001 | 2.12 (1.72-2.63) | <.001 |
| Weight status |  |  |  |  |  |  |  |  |  |  |
| Normal weight | reference |  | reference |  | reference. |  | reference |  | reference |  |
| Underweight | 1.14 (0.72-1.81) | .56 | 1.58 (1.17-2.12) | .003 | 1.77 (1.32-2.38) | <.001 | 1.48 (0.90-2.42) | .12 | 1.28 (0.92-1.79) | .14 |
| Overweight and obesity | 1.23 (0.80-1.90) | .34 | 1.64 (1.21-2.23) | .002 | 1.55 (1.13-2.14) | .007 | 1.44 (0.89-2.34) | .14 | 1.19 (0.84-1.70) | .33 |
| Vitamin D nutritional status |  |  |  |  |  |  |  |  |  |  |
| Sufficiency | reference |  | reference |  | reference. |  | reference |  | reference |  |
| Insufficiency/Deficiency | 1.95 (1.37-2.77) | <.001 | 1.46 (1.12-1.91) | .006 | 1.37 (1.03-1.82) | .03 | 1.73 (1.14-2.63) | .01 | 1.51 (1.13-2.01) | .005 |
