# Supplementary material for: Association of Vitamin D and Weight Status With Neurodevelopmental Outcomes in a Large Pediatric Population: Cross-Sectional Study
Source: JMIR Public Health Surveill. 2026 Feb 27;12:e89756. doi: 10.2196/89756 (PMC12988349; doi:10.2196/89756)
Supplement: Multimedia Appendix 6 [file publichealth_v12i1e89756_app6.docx]

**Multimedia Appendix 6:** Associated factors of being at risk of neurodevelopmental delay in boys under 6 years old by the logistic regression analysis (n=5,794).

| Characteristics | Overall | | | Communication | | | Gross Motor | | | Fine Motor | | | Problem Solving | | | Personal-Social | |
| --- | --- | --- | --- | --- | --- | --- | --- | --- | --- | --- | --- | --- | --- | --- | --- | --- | --- |
|  | OR (95%CI) | *P* value | OR (95%CI) | | *P* value | OR (95%CI) | | *P* value | OR (95%CI) | | *P* value | OR (95%CI) | | *P* value | OR (95%CI) | | *P* value |
| Age | 0.88 (0.83-0.92) | <.001 | 0.66 (0.59-0.73) | | <.001 | 1.00 (0.92-1.08) | | .93 | 0.92 (0.86-0.99) | | .03 | 0.71 (0.63-0.81) | | <.001 | 0.92 (0.85-0.99) | | .02 |
| Weight status |  |  |  | |  |  | |  |  | |  |  | |  |  | |  |
| Normal weight | reference |  | reference | |  | reference | |  | reference | |  | reference | |  | reference | |  |
| Underweight | 1.19 (0.92-1.54) | .18 | 0.95 (0.54-1.66) | | .85 | 1.91 (1.35-2.69) | | <.001 | 1.60 (1.14-2.26) | | .007 | 1.31 (0.72-2.38) | | .37 | 0.97 (0.64-1.46) | | .87 |
| Overweight and obesity | 1.30 (1.01-1.68) | .04 | 1.45 (0.92-2.27) | | .11 | 1.96 (1.38-2.79) | | <.001 | 1.70 (1.21-2.40) | | .002 | 1.55 (0.91-2.64) | | .11 | 1.32 (0.91-1.92) | | .14 |
| Vitamin D nutritional status |  |  |  | |  |  | |  |  | |  |  | |  |  | |  |
| Sufficiency | reference |  | reference | |  | reference | |  | reference | |  | reference | |  | reference | |  |
| Insufficiency/Deficiency | 1.26 (0.99-1.59) | .06 | 1.57 (1.02-2.42) | | .04 | 1.24 (0.87-1.76) | | .24 | 1.19 (0.84-1.68) | | .33 | 1.54 (0.92-2.58) | | .10 | 1.37 (0.97-1.93) | | .07 |
