# Supplementary material for: Association of Vitamin D and Weight Status With Neurodevelopmental Outcomes in a Large Pediatric Population: Cross-Sectional Study
Source: JMIR Public Health Surveill. 2026 Feb 27;12:e89756. doi: 10.2196/89756 (PMC12988349; doi:10.2196/89756)
Supplement: Multimedia Appendix 7 [file publichealth_v12i1e89756_app7.docx]

**Multimedia Appendix 7:** Associated factors of being at risk of neurodevelopmental delay in girls under 6 years old by the logistic regression analysis (n=4,271).

| Characteristics | Overall | | Communication | | Gross Motor | | Fine Motor | | Problem Solving | | Personal-Social | |
| --- | --- | --- | --- | --- | --- | --- | --- | --- | --- | --- | --- | --- |
|  | OR (95%CI) | *P* value | OR (95%CI) | *P* value | OR (95%CI) | *P* value | OR (95%CI) | *P* value | OR (95%CI) | *P* value | OR (95%CI) | *P* value |
| Age | 0.78 (0.73-0.84) | <.001 | 0.62 (0.52-0.75) | <.001 | 0.97 (0.87-1.07) | .54 | 0.73 (0.64-0.84) | <.001 | 0.58 (0.48-0.71) | <.001 | 0.75 (0.65-0.85) | <.001 |
| Weight status |  |  |  |  |  |  |  |  |  |  |  |  |
| Normal weight | reference |  | reference |  | reference |  | reference |  | reference |  | reference |  |
| Underweight | 1.31 (0.87-1.96) | .19 | 1.81 (0.80-4.09) | .16 | 0.97 (0.53-1.78) | .92 | 2.35 (1.32-4.18) | .004 | 1.94 (0.80-4.69) | .14 | 2.55 (1.45-4.48) | .001 |
| Overweight and obesity | 0.88 (0.53-1.47) | .62 | 0.28 (0.04-2.06) | .21 | 1.01 (0.52-1.97) | .97 | 0.91 (0.37-2.28) | .84 | 1.05 (0.32-3.41) | .94 | 0.55 (0.17-1.77) | .32 |
| Vitamin D nutritional status |  |  |  |  |  |  |  |  |  |  |  |  |
| Sufficiency | reference |  | reference |  | reference |  | reference |  | reference |  | reference |  |
| Insufficiency/Deficiency | 2.04 (1.50-2.78) | <.001 | 3.20 (1.75-5.85) | <.001 | 1.93 (1.27-2.93) | .002 | 1.99 (1.18-3.35) | .01 | 2.19 (1.09-4.43) | .03 | 1.91 (1.12-3.26) | .02 |
