# Supplementary material for: Association of Vitamin D and Weight Status With Neurodevelopmental Outcomes in a Large Pediatric Population: Cross-Sectional Study
Source: JMIR Public Health Surveill. 2026 Feb 27;12:e89756. doi: 10.2196/89756 (PMC12988349; doi:10.2196/89756)
Supplement: Multimedia Appendix 8 [file publichealth_v12i1e89756_app8.docx]

**Multimedia Appendix 8:** Associated factors of different CPRS subscales in children above 6 years by the logistic regression analysis (n=9,218).

| Characteristics | Oppositional | | Learning problems | | Psychosomatic problems | | Hyperactivity-Impulsivity | | Anxiety | |
| --- | --- | --- | --- | --- | --- | --- | --- | --- | --- | --- |
|  | OR (95%CI) | *P* value | OR (95%CI) | *P* value | OR (95%CI) | *P* value | OR (95%CI) | *P* value | OR (95%CI) | *P* value |
| Age | 0.94 (0.90-0.98) | .007 | 0.98 (0.95-1.00) | .08 | 0.95 (0.92-0.98) | .003 | 0.97 (0.92-1.03) | .34 | 1.04 (0.98-1.11) | .21 |
| Sex |  |  |  |  |  |  |  |  |  |  |
| Boy | reference |  | reference |  | reference |  | 2.00 (1.44-2.79) | <.001 | 1.85 (1.24-2.75) | .003 |
| Girl | 1.50 (1.21-1.87) | <.001 | 1.63 (1.42-1.87) | <.001 | 1.05 (0.89-1.24) | .59 | reference |  | reference |  |
| Weight status |  |  |  |  |  |  |  |  |  |  |
| Normal weight | reference |  | reference |  | reference |  | reference |  | reference |  |
| Underweight | 0.98 (0.66-1.46) | .94 | 1.15 (0.90-1.46) | .27 | 1.31 (1.01-1.71) | .04 | 1.35 (0.86-2.12) | .19 | 1.44 (0.83-2.51) | .20 |
| Overweight and obesity | 0.90 (0.68-1.18) | .44 | 1.11 (0.95-1.31) | .20 | 0.68 (0.55-0.85) | <.001 | 0.71 (0.49-1.04) | .08 | 1.10 (0.74-1.65) | .64 |
| Vitamin D nutritional status |  |  |  |  |  |  |  |  |  |  |
| Sufficiency | reference |  | reference |  | reference |  | reference |  | reference |  |
| Insufficiency/Deficiency | 1.28 (1.03-1.60) | .03 | 1.40 (1.22-1.61) | <.001 | 1.63 (1.38-1.93) | <.001 | 1.51 (1.13-2.02) | .006 | 1.34 (0.94-1.90) | .10 |
