# Supplementary material for: Association of Vitamin D and Weight Status With Neurodevelopmental Outcomes in a Large Pediatric Population: Cross-Sectional Study
Source: JMIR Public Health Surveill. 2026 Feb 27;12:e89756. doi: 10.2196/89756 (PMC12988349; doi:10.2196/89756)
Supplement: Multimedia Appendix 9 [file publichealth_v12i1e89756_app9.docx]

**Multimedia Appendix 9:** Associated factors of behavior problems in boys above 6 years by the logistic regression analysis (n=5,771).

| Characteristics | Overall | | | Oppositional | | | Learning problems | | | Psychosomatic problems | | | Hyperactivity-Impulsivity | | | Anxiety | |
| --- | --- | --- | --- | --- | --- | --- | --- | --- | --- | --- | --- | --- | --- | --- | --- | --- | --- |
|  | OR (95%CI) | *P* value | OR (95%CI) | | *P* value | OR (95%CI) | | *P* value | OR (95%CI) | | *P* value | OR (95%CI) | | *P* value | OR (95%CI) | | *P* value |
| Age | 0.94 (0.91-0.97) | <.001 | 0.97 (0.91-1.03) | | .26 | 0.99 (0.95-1.02) | | .44 | 0.87 (0.83-0.91) | | <.001 | 0.96 (0.90-1.03) | | .21 | 0.96 (0.88-1.04) | | .30 |
| Weight status |  |  |  | |  |  | |  |  | |  |  | |  |  | |  |
| Normal weight | reference |  | reference | |  | reference | |  | reference | |  | reference | |  | reference | |  |
| Underweight | 1.34 (1.05-1.70) | .02 | 1.10 (0.66-1.82) | | .72 | 1.23 (0.90-1.70) | | .20 | 1.48 (1.08-2.03) | | .02 | 1.29 (0.76-2.18) | | .34 | 1.76 (0.98-3.18) | | .06 |
| Overweight and obesity | 0.92 (0.77-1.09) | .34 | 0.79 (0.55-1.14) | | .21 | 1.15 (0.93-1.43) | | .19 | 0.62 (0.47-0.82) | | <.001 | 0.79 (0.53-1.19) | | .26 | 1.11 (0.70-1.76) | | .66 |
| Vitamin D nutritional status |  |  |  | |  |  | |  |  | |  |  | |  |  | |  |
| Sufficiency | reference |  | reference | |  | reference | |  | reference | |  | reference | |  | reference | |  |
| Insufficiency/Deficiency | 1.49 (1.29-1.73) | <.001 | 1.25 (0.92-1.69) | | .15 | 1.26 (1.05-1.53) | | .02 | 1.70 (1.38-2.11) | | <.001 | 1.44 (1.03-2.01) | | .03 | 1.46 (0.98-2.18) | | .06 |
