# Supplementary material for: Association of Vitamin D and Weight Status With Neurodevelopmental Outcomes in a Large Pediatric Population: Cross-Sectional Study
Source: JMIR Public Health Surveill. 2026 Feb 27;12:e89756. doi: 10.2196/89756 (PMC12988349; doi:10.2196/89756)
Supplement: Multimedia Appendix 10 [file publichealth_v12i1e89756_app10.docx]

**Multimedia Appendix 10:** Associated factors of behavior problems in girls above 6 years by the logistic regression analysis (n=3,447).

| Characteristics | Overall | | | Oppositional | | | Learning problems | | | Psychosomatic problems | | | Hyperactivity-Impulsivity | | | Anxiety | |
| --- | --- | --- | --- | --- | --- | --- | --- | --- | --- | --- | --- | --- | --- | --- | --- | --- | --- |
|  | OR (95%CI) | *P* value | | OR (95%CI) | *P* value | | OR (95%CI) | *P* value | | OR (95%CI) | *P* value | | OR (95%CI) | *P* value | | OR (95%CI) | *P* value |
| Age | 0.97 (0.94-1.01) | .10 | 0.92 (0.86-0.98) | | .008 | 0.96 (0.93-1.00) | | .06 | 1.06 (1.01-1.11) | | .01 | 1.00 (0.90-1.12) | | .94 | 1.27 (1.13-1.43) | | <.001 |
| Weight status |  |  |  | |  |  | |  |  | |  |  | |  |  | |  |
| Normal weight | reference |  | reference | |  | reference | |  | reference | |  | reference | |  | reference | |  |
| Underweight | 1.12 (0.81-1.54) | .50 | 0.84 (0.45-1.58) | | .58 | 1.04 (0.72-1.51) | | .83 | 1.02 (0.63-1.65) | | .94 | 1.55 (0.65-3.71) | | .32 | 0.41 (0.06-3.09) | | .39 |
| Overweight and obesity | 1.05 (0.83-1.31) | .69 | 1.06 (0.70-1.58) | | .79 | 1.07 (0.83-1.38) | | .62 | 0.89 (0.63-1.26) | | .51 | 0.42 (0.15-1.19) | | .10 | 1.32 (0.59-2.95) | | .50 |
| Vitamin D nutritional status |  |  |  | |  |  | |  |  | |  |  | |  |  | |  |
| Sufficiency | reference |  | reference | |  | reference | |  | reference | |  | reference | |  | reference | |  |
| Insufficiency/Deficiency | 1.64 (1.36-1.97) | <.001 | 1.35 (0.98-1.88) | | .07 | 1.59 (1.29-1.96) | | <.001 | 1.44 (1.10-1.90) | | .009 | 1.71 (0.93-3.18) | | .09 | 0.88 (0.42-1.84) | | .73 |
